# Acceptability and feasibility of a faculty development programme for medical and dental academics in Ghana

**DOI:** 10.1186/s12909-026-08826-3

**Published:** 2026-02-21

**Authors:** A. B Konadu, E. N.Y Nyarko, K. Asah Opoku, D. Tormeti, A.B Hottor, A. Drovandi, E.A Yawson, S. Hewlett, D.N Banyubala, J. Hart, S. Conen, D.D Siriwardhana, S. Harris, J. Grundy, L. Byrne-Davis

**Affiliations:** 1 University of Ghana Dental School, College of Health Sciences, University of Ghana, Accra, Ghana; 2 University of Ghana Medical School, College of Health Sciences, University of Ghana, Accra, Ghana; 3 School of Medical Sciences, Faculty of Biology, Medicine and Health, University of Manchester, Manchester, M13 9PL UK; 4 Medical and Dental Council of Ghana, Accra, Ghana

**Keywords:** Continual professional development, Dentistry, Medicine, Mixed-methods, Perceptions

## Abstract

**Background:**

Universal health coverage is more likely to be achieved by competency-based education of health workers than by traditional, time-based learning. Large-scale transitions in pedagogical approaches to such health worker education require effective faculty development. This research aimed to examine the feasibility and acceptability of a UK-based faculty development programme tailored for medical and dental educators within the Ghanaian context.

**Methods:**

A series of faculty development workshops was provided by a UK-based medical programme, focussing on facilitating the transition from didactic teaching to competence-based medical and dental education. Perceptions of feasibility and acceptability were captured from participants through a post-intervention survey and semi-structured interviews, both utilising the Theoretical Framework of Acceptability, and analysed using thematic analysis.

**Results:**

From 27 survey and 15 interview participants representing leaders in medical and dental education in Ghana, the faculty development workshops were positively received, perceived as effectively delivering pedagogical content relevant to medical and dental education in a way that was both engaging and applicable to the local context. Participants felt confident in applying the taught concepts such as clinical placement supervision and clinical reasoning, as well as confident in leading broader uptake of competency-based medical and dental education across educational institutions in Ghana. Conflicting priorities and high workloads for medical and dental educators were perceived as the main barriers to participation and uptake of the workshops nationally.

**Conclusion:**

Continuing professional development with contemporary pedagogical approaches is considered essential by educators for training the next generation of health professionals.

**Supplementary Information:**

The online version contains supplementary material available at 10.1186/s12909-026-08826-3.

## Introduction

Universal health coverage (UHC) requires all people have timely access to high-quality and affordable healthcare [1], achieved through a workforce of competent, versatile, and ‘geo-glocal’ health professionals [2]. Educating an effective health workforce to meet UHC requires adoption of innovative, evidence-based curricula for both students and educators, and the application of novel teaching techniques during their training [3]. Medical and dental education specifically are experiencing constant evolution [4], arising through rapidly expanding volumes of clinical knowledge within a highly regulated environment with strict professional standards [5]. These requirements of expanding knowledge transfer create significant hurdles for medical and dental educators and learners. A complicating factor of knowledge growth is the concurrent shift from ‘traditional’ didactic-based instruction to experiential and competency-based learning models [6]. 

Competency-based medical and dental education (CBMDE) represents one such shift in the pedagogical norm, utilising an outcomes-oriented framework for designing, implementing, and evaluating educational programmes, as well as assessing learners throughout their training continuum. It is rooted in measurable competencies that focus on the clinical performance of healthcare professionals [7], amalgamating experiential learning and practical application allowing students to develop both theoretical knowledge and ‘hands-on’ skills simultaneously [8, 9]. 

### Local context

The healthcare system in Ghana, like many African counterparts, is under increasing strain to address the complex health needs of its population. Healthcare infrastructural deficits coupled with a rising prevalence of non-communicable diseases warrants more effective and versatile healthcare professionals [10, 11]. These professionals require stronger clinical skills founded on basic, pre-and para-clinical sciences, adaptability, and critical thinking essential for navigating a rapidly changing landscape. Delivering an education that enables adaptation to these changes demands a collaborative national effort, including faculty development, to transform curricula and assessment. In high-income countries, such as the United Kingdom and United States, structured faculty development programmes are often well-established within medical and dental programmes. The University of Manchester’s ‘Professionals in Medical Education’ (PRiME) faculty development programme, for example, offers comprehensive education and training through various components, supporting thousands of educators in their continual professional development [12]. Such programmes have proven effective in enhancing teaching quality and meeting regulatory requirements.

The Medical and Dental Council (MDC) of Ghana has partnered with the University of Manchester and the University of Ghana to apply the PRiME faculty development workshops for Ghana’s medical and dental educators. This research aimed to explore the feasibility and acceptability of these workshops tailored to the Ghanaian context, and how a competency-based approach could enhance existing teaching and learning frameworks. The research questions were: (1) How feasible and acceptable are tailored UK faculty development workshops within the Ghanaian context for medical and dental educators? and (2) What enablers and barriers are perceived as influencing the implementation of competency-based medical and dental education in Ghana?

## Methods

### Design

This was a mixed-methods study using a post-intervention survey and semi-structured interviews of senior medical and dental education leaders in Ghana. The study is reported according to the COnsolidated criteria for REporting Qualitative Research (COREQ) checklist [13], and the Checklist for Reporting Results of Internet E-Surveys (CHERRIES) [14]. 

### Ethics

Ethical approval was obtained from the Ethical and Protocol Review Committee (EPRC) of the College of Health Sciences (CHS), University of Ghana (CHS-Et/M.1-P4.19/2024–2025).

### Participants

Eligible participants were medical and dental educators teaching at Ghanian public and private medical and dental schools, and senior leadership of these schools with roles relating to teaching design and implementation.

### Setting and intervention

Specific workshops from the University of Manchester PRiME suite were chosen by clinical academics at the University of Ghana (ABH, EAY, SH) and MDC-affiliated (DNB) authors for tailoring and presentation by the University of Manchester-affiliated authors (JH, SH, JG, LBD). Details of these workshops are available in Table 1, which were delivered to participants over two consecutive days in November 2024. Workshops consisted of presentations, case scenarios, and related facilitated discussions to help participants apply theory and learning to their context.

Participants were first introduced to the broader PRiME ethos and educational approach, followed by discussion of the World Health Organisation ‘Global Competency Framework’ and broader rationale for CBMDE [15], including curricula, implementation considerations and logistics, and how this relates to teaching and assessment standards, and clinical placement supervision. The second day of workshops introduced CBMDE-specific teaching approaches aligned to the theory and rationale for clinical reasoning teaching and assessment. There was also dedicated time for group discussions to consolidate concepts and apply principles learned during the workshops to individual and local context.

**Table 1:** Content of the four professionals in medical education: PRiME workshops delivered to Ghanaian medical and dental educators

Session Title	Content
Introduction to the PRiME Staff Development Programme	This workshop included a description of the approach and best-evidence in health professional education and pedagogical approaches used for development, design and delivery of the UK PRiME Programme, which has an overall aim of supporting and valuing colleagues in their continuing professional development. There was explanation of the structure and ethos of PRiME, with PRiME Core components preparing individuals ahead of taking on specific teaching roles, PRiME Advanced available to develop further as a clinical teacher and educator, and PRiME Network to bring people together to share experience and ideas.
PRiME Core: Competency Based Medical and Dental Education	This workshop was an introduction to the WHO frameworks and rationale for Competency Based Medical and Dental Education (CBMDE). Applications of CBMDE in relation to pedagogy and assessment were outlined, with facilitated discussion to aid participant understanding of the key terminology and concepts in relation to Universal Health Coverage.
PRiME Core: Clinical Placement Supervision	This workshop outlined the role of a clinical placement supervisor, and how colleagues are prepared and trained for this role at The University of Manchester, UK. Concepts were discussed in the context of CBMDE, and facilitated discussion allowed application to the Ghanaian context.
PRiME Advanced: Teaching Clinical Reasoning	This workshop introduced participants to the theory and rationale for use of clinical reasoning approaches in medical and dental education, including assessment. Participants were invited to practice a selection of recognised teaching methods using clinical case studies, with facilitated discussion to apply concepts to the Ghanaian clinical setting and workplace. There was then time dedicated to consideration of all the aspects discussed in the preceding workshops in the context of participants’ own workplaces, and as could be applied in Ghana.

### Post-workshop survey

The survey was composed of demographic and professional experience questions (gender identity, age, educational role, and years of experience), followed by Likert-scale and paired open-ended questions aligning with the ‘Theoretical Framework of Acceptability’ (TFA) generic questionnaire [16]. The TFA questionnaire asks questions aligned to seven specific elements (affective attitude, burden, ethicality, perceived effectiveness, intervention coherence, self-efficacy, opportunity costs) and ‘general acceptability’. The survey (see Appendix 1) was developed by the UK authors and pre-tested by Ghanaian members of the research team, with minor wording adjustments made to improve comprehensiveness. An information and consent page preceded the survey questions, with consent implied by submission of a completed survey.

### Semi-structured interview schedule

The semi-structured interviews were also developed and tested by the UK and Ghanian authors respectively. Questions focused on educational experiences, engagement in professional development activities, acceptability of the PRiME workshops, and feasibility of their tailoring and implementation more broadly across medical and dental education in Ghana (Appendix 2).

### Procedure

A purposive sampling approach was used, coordinated by Ghana’s MDC, who contacted each medical and dental school requesting distribution of the invitation to participate to their leadership and teaching staff. Participants expressing interest were then invited to participate to ensure spread of seniority and teaching experience. All participants received an information sheet and had the opportunity to ask questions before providing informed written consent. This approach adhered to established frameworks for evaluating complex educational interventions in resource-limited settings and allowed for rapid and frequent feedback while maintaining methodological rigor [17]. 

A QR code was provided for the post-workshop survey, and all participants were emailed an invitation to participate in an interview. Interviews were audio-recorded, lasting between 25 and 40 min, with field notes taken during each interview. Interviews were conducted by Ghanaian authors ABK (female, BDS), ENYN (male, PhD), and KAO (male, PhD), who were trained by an experienced qualitative researcher (author AD). Repeat interviews were not conducted, and transcripts were not returned to participants for comment or clarification. No incentives were offered for participation in the workshops, survey or interview,

### Data analysis

Quantitative survey data from the demographic and TFA-aligned Likert-scale questions were summarised and presented using descriptive statistics. Free text responses related to acceptability and feasibility were analysed using conceptual content analysis [16]. Interview transcripts were checked for accuracy, and all identifying information was redacted. Anonymized transcripts were analysed using the thematic analysis approach advocated by Braun and Clarke (2006) [18], mapping to the specific elements of the TFA. Analyses were undertaken by authors ABK, ENYN and KAO, who independently familiarized themselves with the transcripts and coded using a line-by-lone open coding process, with themes generated through a constant comparison process as advocated by Corbin and Strauss (2014) [19]. These three authors then met to discuss findings, review their generated codes and themes, and come to consensus on regrouped themes and subthemes to align with the TFA and its specific elements. This was followed by discussion and confirmation across the entire research team during a workshop in Ghana in February 2025. Mapping of data to the TFA, and triangulation between the survey Likert-scale and open-ended responses, and interviews was used to understand the findings more comprehensively [20]. This approach involved ensuring consistent interpretation of each element of the TFA (which are described in the results section) across all authors, followed by point-by-point agreement on which element each sub-theme from the survey and interviews sat within, including associations with other themes and other elements. Perceived overlap in element interpretation and applicability (e.g. burden and opportunity costs) necessitated merging of elements in the results to promote more interpretable findings. Illustrative quotes by survey (S) and interview (I) participants that demonstrate the key themes are reported verbatim to support the findings.

## Results

### Participants

**Fig. 1:**
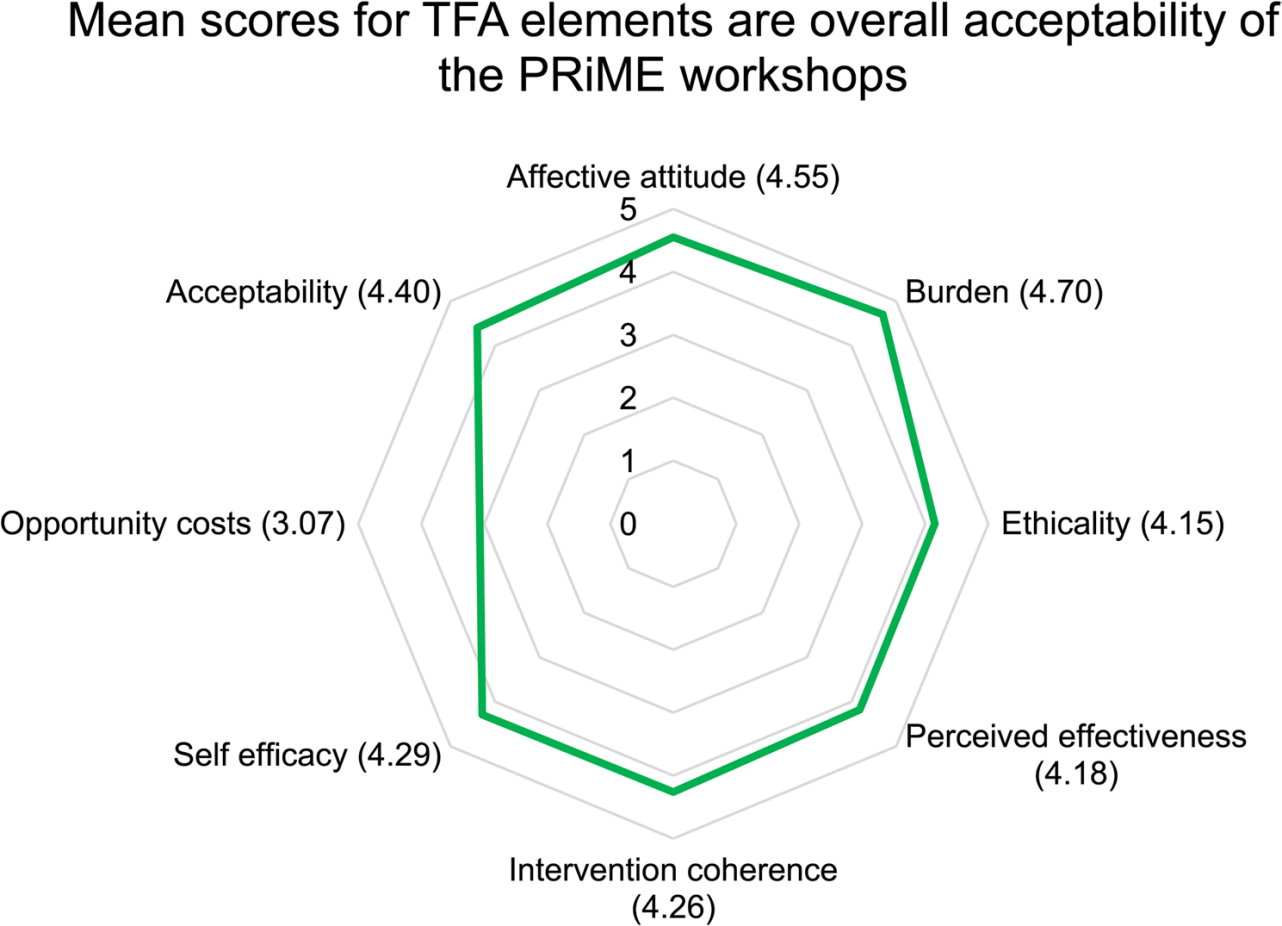
Mean Likert-scale scores for elements of the TFA for the PRiME workshops

Twenty-seven participants attended the workshops and completed the post-workshop survey. Nearly two-thirds of participants were female (16; 59%), with most participants between the ages of 40 and 54 years old (20; 74%). Two-thirds were medical faculty members (17; 63%), with a smaller number of dental faculty members (6; 22%), and those in other senior leadership positions (4; 15%). Most participants also had more than 10 years of experience in higher education, with 11 (41%) with 11–20 years of experience, and 14 (52%) with more than 20 years of experience. Current teaching roles were also varied, across pre-clinical and clinical years of undergraduate medical and dental education as well as postgraduate medical education, using a combination of didactic teaching, simulation, case-based learning and problem-based learning. Characteristics of the 15 interview participants are detailed in Table 2 below.Findings of the survey and interviews are presented aligning with the specific elements of the TFA (affective attitude, burden, ethicality, perceived effectiveness, intervention coherence, self-efficacy, opportunity costs and overall acceptability). From the survey, mean scores (of a maximum of 5) for the Likert-scale questions on each element of the TFA are illustrated in Fig. 1 and detailed in Appendix 3.

**Table 2:** Characteristics of the interview participants (*n* = 15)

ID No./Designation	Gender (F-Female M-Male)	Current Role(s) in Teaching/Learning
I1, Senior Lecturer / HoD	F	Teaches clinical year medical and graduate entry students (didactic teaching, cases, and problem-based learning)
I2, Senior Lecturer / HoD	M	Teaches clinical year medical and graduate entry students (didactic teaching, cases, and problem-based learning), and postgraduate masters-level students
I3, Lecturer	F	Teaches final-year dental students and medical residents (including research skills)
I4, Senior Lecturer / VP	M	Teaches medical students using didactic, clinical, and theatre-based teaching
I5, Dean	M	Teaches pre-clinical undergraduate medical students
I6, Professor / HoD	M	Teaches all levels of undergraduate medical students
I7, Senior Lecturer Coordinator	F	Teaches junior and senior clerkship students and medical residents, and coordinates teaching and learning for a medical school.
I8, Senior Lecturer/ HoD	M	Teaches pre-clinical medical students, and postgraduate masters students
I9, Professor	M	Teaches pre-clinical and clinical years medical students
I10, Senior Lecturer / HoD	M	Teaches dental students through didactic teaching, clinical simulation, and student presentations
I11, Senior Lecturer	F	Teaches medical students using competency-based medical education, and problem based learning
I12, Senior Lecturer	M	Teaches all levels of medical students through didactic lectures, simulation, hands-on-theatre observations and clinical examinations
I13, Professor / HoD	M	Teaches pre-clinical medical students using didactic lectures, problem-based learning, case studies, and student presentations
II4, Professor / HoD	M	Teaches undergraduate medical students using didactic lectures, tutorials, case studies, and coordinates training for surgical units
I15, Professor / Dean	F	Small teaching role, and coordinates modules across the medical school

Specific fields of teaching have been removed to preserve anonymity

*HoD* Head of Department, *I* Interviewee, *VP* Vice President

### Affective attitude

This element describes perceptions about the intervention from an emotional viewpoint before and after participation. Prior participation, some participants indicated scepticism of the value of the workshops and low expectations surrounding applicability of the content.

“I wasn’t very keen, you know? I was like, okay, it’s great to be in a room of medical educators. But I was like, maybe it’s going to be ‘another thing’.” (I11).

“When it was advertised, it doesn’t sound attractive, but when you attend, you realize that really is a lot of deficit.” (I12).

Some of these perceptions appeared to be because of previous workshop experiences that did not meet expectations, and the frustration of limited continuing professional development acknowledgement for attendance. There were no instances of participants expressing excitement, enthusiasm or anticipating enjoyment of the workshops prior participation, which may also be linked to the burden and opportunity costs (see next sub-section of the results).

“I’ve been running CPDs [continuing professional development] for teachers. However, they seem to get very low points… so I was almost discouraged from running them.” (I11).

Despite this lack of positive attitudes towards the workshops prior participation, many participants post-participation described the workshops using positive descriptors such as enjoyable, engaging, encouraging and affirming.

“Nobody held back, and so we’re able to learn more and more and more. I really did enjoy the expertise we had” (I2).

“This hit to the core….I basically identified with this workshop” (S13).

The workshops were also considered reassuring through confirming the appropriateness of teaching methodologies already employed by participants, and serving as a method for examining consistency in teaching methods used by different educators.

“It’s been exciting for me to see that there’s a systematic way of getting all faculty members to have the same kind of vision in terms of the outputs that we want with our students” (I1).

“Historically, we have done rote learning and learning by list, so moving away from that is been very, very good. In my medical training, I did problem-based learning as the core foundation for training. So it’s nice to see it again, and reinforces what I’ve learned” (I4).

### Burden and opportunity costs

Burden is the perceptions of participants on the amount of effort required to participate, while opportunity costs is the extent to which participants feel they must sacrifice benefits, profits or values in order to participate. These two elements are described together due to their overlap in interpretation.

Many participants highlighted competing priorities that had to be rescheduled or missed in order to participate in the workshops, particularly as they ran over two days. These included clinical and academic responsibilities, seen to be made more difficult by their senior positions.

“One of the clashes for this [the workshops] is that it’s running at the same time as another major college meeting” (I4).

“Most people are very busy, so spending two days in a workshop…may not be very easy for them to do it, even as a head of department, where you are still doing the same amount of teaching like you do when you were not a head of department” (I11).

Compounding these conflicting priorities were the associated costs of participation such as transportation and accommodation, and considerations over the parties responsible for paying. These same costs for participating were also forecast as a likely barrier for the implementation of these workshops across the Ghanaian medical and dental higher education sector.

“Bringing people together in a place where you know that if it’s not on the university campus and you’re going out, you need to pay for the venue, provide the news and so on. So that is probably what would cost us something. And if we are bringing facilitators from elsewhere, then maybe the transportation.” (I1).

“Who bears the cost? It has to be the institutions, so the medical schools that maybe would, with support from a regulatory body” (I6).

Conversely, the actual burden of participation was perceived as being low, which was also reflected in the survey, having the highest rating. The level of engagement and other positive elements under ‘affective attitude’ combined with not requiring any pre-work likely contributed to these low perceptions of burden. However, some participants indicated burnout associated with participating.

“So I travelled as an external examiner to go and examine. I returned Sunday evening, and then had to be in this meeting” (I11).

As a comparison to the PRiME workshops, participants stated that other professional development activities such as these were either not available or rarely available, with attendance similarly affected by high workloads and competing priorities. Attendance of these were also sometimes considered as being through a sense of obligation.

“I don’t think that there are any proper structured programs…there are few ad-hoc programs that come up from the Ghana college or from medical school, but it’s not like a program that you know you have to go through” (I7).

“So occasionally you get workshops being organized, and you look at what you think goes to you, and you attend. It’s all usually quite voluntary” (I1).

### Ethicality and perceived effectiveness

Ethicality relates to how participants perceive the intervention as being aligned with their own system of values, within this context relating to professional duties and institutional responsibility, beliefs on what constitutes good teaching and learning, and alignment with improving patient care and health outcomes through producing competent medical and dental graduates. These perceptions are inherently linked to perceived effectiveness, the element which describes participant perceptions of how likely the intervention is in achieving its expected purpose.

Participants highlighted the responsibility of themselves and their institutions to teach the next generation of clinicians who are confident and competent in their respective practices, which includes implementing innovative educational approaches such as those discussed in the workshops.

“I think it’s very innovative, very useful and very timely, because the world is evolving and from time to time, we would need to also evolve as well in our style of teaching” (I9).

“We feel an obligation to teach the next generation…we want them to be competent. So, I’m sure every medical educator is looking at how they can improve their teaching” (I1).

Specific content which participants felt aligned with achieving this goal included developing improved clinical reasoning for students, and assessments which were fair and more objective, with transparency and accountability in how they were delivered and marked.

“It’s just reinforced my goals for teaching and also my style. And I think that it’s encouraged me to continue to get the students to move beyond just recall to thinking and reasoning and developing that skill” (I2).

“the assessment can be a bit subjective, and that is why these OSCEs and other things come in to, I mean, take away the level of subjective” (I4).

“The clinical exams…move away from simple recall kind of questions to scenario based, that tests higher order things. And then with the with the clinical exams, you can say we basically are now covering all the objectives of the course, so the stations are match to the objectives of the course”. (I13)

The concept of facilitating adult learning and effectively ‘teaching the teacher’ was widely referenced by participants, including the confirmation of existing teaching approaches and introduction of new approaches considered beneficial for student learning.

“I loved the pace, the interactions - small group discussions; reflections, activities - and they were well interspersed. Truly engaging for everyone” (S18).

I have fairly good knowledge in that space. But in this workshop, I’ve learned new things. New concepts have come up I wasn’t aware of so overall, it’s been very good” (I12).

Suggestions for potential improvements in the workshops for the Ghanaian context included Ghanaian-specific examples of applications, and workshop elements tailored to the different stage of medical and dental education; pre-clinical, para-clinical and clinical.

“The suggestion will be that it should be sub-tailored, also in case of future training, to look at specific disciplines, so we can look at, probably getting basic sciences, targeting pre-clinical or para clinical, targeting the clinical etcetera” (I2).

### Intervention coherence and self-efficacy

Intervention coherence is the extent to which a participant understands the structure and function of the intervention, while self-efficacy describes their confidence in performing behaviours required to participate in and after the intervention. These elements are described together as the second relies on the first, and below also includes participant perceptions on their capacity to rollout the PRiME workshops in their respective institutions.

Many participants felt that CBMDE was at least partially incorporated into their standard teaching approach, indicating that terminology differences were discussed within the workshops. For those with experience in CBMDE, they felt it reassured the approaches they had already been taking.

“I think by and large, we do a competency-based training. It’s just, we don’t really call it that” (I1).

“I would say that all of a sudden we have to put a definition that we do, which we have done, without putting definition” (I12).

Despite this, there was broad acknowledgement of the value of discussions on existing teaching methods as well as new teaching methods.

“It will help us to look at our curriculum again…looking at how we can incorporate certain things like problem-based learning, PBLs, you know, into our curriculum” (I14).

“I can see that we are moving forward to a different way of teaching, from our traditional ways to, you know, the new way of teaching” (I3).

“It has been a great two days that has exposed me to some concepts that underpin CME” (S21).

Participants felt confident in personally adopting the approaches covered in the workshop and adapting their personal teaching style to integrate these practices. There were also considerations as to how the CBMDE approach fits into existing curricula and systems, and supporting more widespread adoption of the principles from the workshops including input from the regulator.

“Easily, because I already have solid foundation in this area” (I13)

“I think it's doable, once we have the resources. We have key people who are trained. So, it's like, train the trainer, isn't it? So, once you have a few people who are trained, then you can, you can perpetrate it and spread it.” (I4)

“I think MDC can make it one of their CPD-credited programs, and should be regional based or zoned – say we can have a south and northern zone trainings. If they do that, I think a lot more people will join” (I15)

“As a dean, I have that that power to cause curriculum development, and then if try to push some of these changes into the program and into the faculty,” (I5)

Self-efficacy challenges, however, were also acknowledged, with an emphasis on resource limitations, including finances, time and infrastructure, as well as a perceived generational gap within the faculty and expected disinterest in adjusting teaching approaches.

“It’s still the same things, time, the structure of the institution, the personnel work, the number of students they have to work with, I think, and resources” (I11).

“If we are bringing facilitators from elsewhere, then maybe the transportation, accommodation and other catering needs may contribute towards the cost” (I1).

“It is difficult. It will take time, because our resources are not as in other parts of the world, so it will be challenging to implement” (I3).

## Discussion

This study examined the acceptability and feasibility of a tailored suite of faculty development programme for medical and dental educators in Ghana, and barriers to the implementation of competency-based education. Participants included deans, and medical and dental educators of different seniority and experiences, with broad educational and training responsibilities including teaching, simulations, demonstrations, supervision, and the delivery of clinical and surgical instructions to rotating students. Perceptions across most elements of the TFA were very positive and consistent across the survey and interviews, with the faculty development workshops viewed as engaging, effective, aligned to the needs of medical and dental education in Ghana, and considered feasible to implement for educators. Perceptions of the main barriers to both participation in the workshops and implementation of their content were competing priorities and high workloads of Ghanaian clinicians and educators, and the financial costs associated with broader implementation.

Medical and dental education in Ghana began with the apprenticeship model, followed by mentorship, and has since evolved into the current system, incorporating both mentorship and the formal integration of clinical clerkships within a university framework [21]. This aligns with other research indicating the global need for using modern pedagogical approaches for training educators [22, 23]. This study indicates medical and dental educator receptivity to the next shift in education to being competency-based, facilitated through structured faculty training workshops. The perceived lack of opportunities for formal training courses in Ghana (as is more common in the Global South) [24] likely contributed to the positive perceptions of the workshops, and supports breaking the cycle of ‘teaching in the same manner in which you learned’ [21, 24]. The adoption of such faculty development however requires ‘translation’ to the local context. The faculty development programme deliverers were from the UK. This study did not explore the perspectives of the participants in terms of who should deliver this type of education and training, and whether having international educators with a lot of experience is beneficial or not. The UK has an increasingly international outlook in terms of higher education, with increasing numbers of branch campuses and transnational education delivery. Future studies should explore the acceptability, feasibility and effectiveness of this type of so-called ‘flying faculty’, in addition to educational content.

Even if training is perceived as acceptable, implementing the knowledge and skills gain in work can be difficult, with these sorts of behaviour changes influenced by the opportunities (both physical and social) in the working environment [25]. Some participants indicated readiness to personally implement the knowledge and skills acquired, while some further reported readiness to pass on training to colleagues. Others however highlighted the opportunity costs of faculty training, indicating concerns of large student numbers and existing high workload of academics and clinicians, lacking infrastructure, and systemic ‘will’ to support broader development of the skills of teaching (e.g. PBL). Such concerns have been documented elsewhere, particularly in low resource training settings, including in nursing education [26]. These limitations require the innovative use of resources, including online resources and student/faculty pre-training on these innovative pedagogies, as means of effectively implementation despite logistical challenges [27]. 

Further, buy-in from all stakeholder groups, such as regulators, in this case including the MDC and the Ghana Tertiary Education Commission are required for the successful adoption of contemporary pedagogical practices. Support from such groups were perceived as major facilitators, through appropriate policy support and accreditation of learning for CPD [28], as well as financial governmental and state support. Support from the central administration of the institutions of the medical and dental schools were also raised.

Strengths of this study include the use of the TFA as an internationally recognised model of examining acceptability aligned to multiple methods of data collection, and high participation rate across the survey (100%) and interviews (56%). Limitations include a relatively small number of workshop (and therefore survey) participants which may not accurately reflect the broader population of medical and dental educators, and the cross-sectional nature of the intervention and gathering of perceptions rather than a continuous process. Another potential limitation is that some of the researchers (JH, LBD, SC, SH, JG) were also the deliverers of the faculty development programme. This might have introduced a social desirability bias with the participants having met the research team. We mitigated for this in the survey data by ensuring surveys were anonymous. The interviews, which were obviously not anonymous, were conducted by the other researchers to mitigate for lack of anonymity. Transcripts were anonymised prior to analysis by the wider team and participants were assured of this level of anonymisation. All researchers are active in the teaching and learning of health professionals and this will have influenced analyses of the data. Mitigation of the impact of this on the findings presented in this paper was attempted by reflexivity. To this end, all researchers critically examined how our experiences as educators influenced our decisions in both the creation of the survey and interview guide and the interpretation of the data collected. Analyses were discussed by the researchers in light of our position as educators. Through the acknowledgement of our own positionality we sought to enhance participant perspectives in our analyses. Nonetheless, the results should be interpreted in awareness of both the potential desirability bias of the participants, and the positionality of the researchers.

## Conclusion

This study highlights the demand and feasibility of implementing a faculty development programme within the Ghana. Enhanced teaching methodologies and the adoption of innovative pedagogical techniques were seen as key positives, while significant challenges were also raised, such as funding constraints and infrastructural limitations. Therefore, embedding continuing professional development in medical and dental education as a core requirement in teaching institutions is likely to be acceptable and perceived as effective, but its feasibility will be contingent on overcoming significant challenges.

## Supplementary Information

Supplementary Material 1.

Supplementary Material 2.

Supplementary Material 3.

## Data Availability

Materials, data available from corresponding author on reasonable request and according to ethics permission.

## Abbreviations

CBMDE: Competency-Based Medical and Dental Education
CHS: College of Health Sciences
CHERRIES: Checklist for Reporting Results of Internet E-Surveys
CPD: Continuing Professional Development
COREQ: COnsolidated criteria for REporting Qualitative Research
EPRC: Ethical and Protocol Review Committee (EPRC)
MDC: Medical and Dental Council
PBL: Problem-Based Learning
PRiME: Professionals in Medical Education
UoM: University of Manchester
UK: United Kingdom
