# Supplementary material for: Acceptability and feasibility of a faculty development programme for medical and dental academics in Ghana
Source: BMC Med Educ. 2026 Feb 21;26:506. doi: 10.1186/s12909-026-08826-3 (PMC13032255; doi:10.1186/s12909-026-08826-3)
Supplement: Supplementary file 1 — Supplementary Material 1. [file 12909_2026_8826_MOESM1_ESM.docx]

**Appendix 1**

**Questionnaire to assess the acceptability of the tailored suite of PRiME faculty training workshops**

**Guidance:** The survey will take 10-15 minutes to complete, with informed consent implied by submitting a completed survey. The survey will be preceded by an information sheet detailing the purpose of the survey, investigators' contact details, and other required details.

**Questions:** All questions have been set up as forced (mandatory) responses in Qualtrics to increase the item response rate and minimize the missing data. However, participants can proceed to the next question by clicking ‘prefer not to say’ should they wish to do so for any question/s in the survey.

**Part 1: Participant Information**

1. What is your gender identity?
   - Woman
   - Man
   - Non-binary
   - Prefer not to say
   - Other

if other, please add your gender identity here if you like: [free text]

- - Prefer not to say

1. What is your age? [free text]
2. What is your current role in medical or dental education?
   - Practicing medical doctor
   - Practicing dentist
   - Faculty member – Medicine – please specify your designation [free text]
   - Faculty member – Dentistry – please specify your designation [free text]
   - Prefer not to say
   - Other – please specify [free text]
   - Prefer not to say
3. How many years of experience do you have in this current role? ______________years [free text]
4. How many years of work experience do you have in total? ______________years [free text]
5. In which country did you do your undergraduate training? [free text]
6. Please mark your participation status (attendance) in the following staff development workshops:

| Workshop | Participation status | | | |
| --- | --- | --- | --- | --- |
|  | Full participation | Partial participation | No participation | Prefer not to say |
| Workshop 1 |  |  |  |  |
| Workshop 2 |  |  |  |  |
| Workshop 3 |  |  |  |  |
| Workshop 4 |  |  |  |  |

**Part 2: Theoretical Framework of Acceptability Generic Questionnaire to assess the acceptability of the tailored suite of PRiME faculty training workshops (with pilot testing and rewording to improve comprehension)**

1. Did you like or dislike this suite of faculty training workshops?

| Strongly dislike | Dislike | Neutral (neither like or dislike) | Like | Strongly like |
| --- | --- | --- | --- | --- |
| 1 | **2** | **3** | **4** | **5** |

- - Prefer not to say

Do you have anything else you would like to mention relating to the question above?………………………………………………………………………………

1. How easy was it to engage with these faculty training workshops?

| Very difficult | Somewhat difficult | Neutral (neither easy or difficult) | Somewhat easy | Very easy |
| --- | --- | --- | --- | --- |
| 1 | **2** | **3** | **4** | **5** |

- - Prefer not to say

Do you have anything else you would like to mention relating to the question above?………………………………………………………………………………

1. How well did the workshops align with your personal values relating to teaching?

| Not aligned | A little aligned | Somewhat aligned | Mostly aligned | Completely aligned |
| --- | --- | --- | --- | --- |
| 1 | **2** | **3** | **4** | **5** |

- - Prefer not to say

Do you have anything else you would like to mention relating to the question above?………………………………………………………………………………

1. This suite of faculty training workshops has improved my teaching competencies:

| Strongly disagree | Disagree | Neutral (neither agree or disagree) | Agree | Strongly agree |
| --- | --- | --- | --- | --- |
| 1 | **2** | **3** | **4** | **5** |

- - Prefer not to say

Do you have anything else you would like to mention relating to the question above?………………………………………………………………………………

1. It is clear to me how these faculty training workshops will help improve my teaching:

| Strongly disagree | Disagree | Neutral (neither agree or disagree) | Agree | Strongly agree |
| --- | --- | --- | --- | --- |
| 1 | **2** | **3** | **4** | **5** |

- - Prefer not to say

*Do you have anything else you would like to mention relating to the question above?……………………………………………………………………………………*

1. How confident did you feel about engaging with these faculty training workshops?

| Very unconfident | Unconfident | Neutral (neither unconfident or confident) | Confident | Very confident |
| --- | --- | --- | --- | --- |
| 1 | **2** | **3** | **4** | **5** |

- - Prefer not to say

Do you have anything else you would like to mention relating to the question above?………………………………………………………………………………

1. Engaging in these faculty training workshops interfered with my other priorities:

| Strongly disagree | Disagree | Neutral (neither agree or disagree) | Agree | Strongly agree |
| --- | --- | --- | --- | --- |
| 1 | **2** | **3** | **4** | **5** |

- - Prefer not to say

Do you have anything else you would like to mention relating to the question above?………………………………………………………………………………

1. How acceptable was this suite of faculty training workshops to you?

| Completely unacceptable | Unacceptable | Neutral (neither unacceptable or acceptable) | Acceptable | Completely acceptable |
| --- | --- | --- | --- | --- |
| 1 | **2** | **3** | **4** | **5** |

- - Prefer not to say

Do you have anything else you would like to mention relating to the question above?……………………………………………………………………………

**Part 3) Additional information on perceptions of acceptability of the PRiME workshops**

1. In what ways were the PRIME staff development training workshops different to training events you have previously completed?

……………………………………………………………………………

1. Please feel free to use this space to expand on any of your ratings above relating to the acceptability of the faculty training workshops.

……………………………………………………………………………
