# Supplementary material for: Acceptability and feasibility of a faculty development programme for medical and dental academics in Ghana
Source: BMC Med Educ. 2026 Feb 21;26:506. doi: 10.1186/s12909-026-08826-3 (PMC13032255; doi:10.1186/s12909-026-08826-3)
Supplement: Supplementary file 2 — Supplementary Material 2. [file 12909_2026_8826_MOESM2_ESM.docx]

**Appendix 2**

**Semi-structured interview guide to evaluate the feasibility of the tailored suite of PRiME faculty training workshops**

Note: blue text will be used exclusively for interviews with medical faculty, and red text will be used exclusively for interviews with dental faculty

**Purpose:** This semi-structured interview aims to gather information on the feasibility of the tailored suite of PRiME faculty training workshops held on [insert Dates] in Ghana. Regarding feasibility, we would like to know your views on to what extent these workshops can be successfully carried out in Ghana. This is expected to inform the development of a faculty training programme for medical and dental educators in Ghana.

**Participants:** Educators from Medical and Dental Schools in Ghana who attended the tailored suite of PRiME faculty training workshops held on [insert Dates] in Ghana are invited to participate in this study.

**Guidance:** This interview will take up to 40-60 minutes and will be audio recorded. The interview will be a one-on-one conversation between a study coordinator and a single participant. Each main question will be asked with sufficient time for the participant to answer, with prompts allowed to support the participant in providing their own opinions on the topic. Questions do not need to be asked strictly in the order they are presented below if a participant provides valued insights earlier on in the question order.

**Preamble for participants:** Thank you for taking the time to participate in this interview. We are interested in your views and ideas on the feasibility of conducting the tailored suite of PRiME faculty training workshops. Just to reiterate there are no right or wrong answers to the questions that will be asked, we are simply interested in your valued opinion.

1. To begin with, could you please provide a brief overview of your current role?

*Prompts if needed:*

- *What type of teaching and learning activities are you involved in?*

1. How do medical (dental) educators in Ghana currently engage in professional development activities?

*Prompts if needed:*

- *What type of training and development activities are available, duration, and where?*
- *Have you undertaken any training and development activities while you are on the job?*

1. Could you tell me about your thoughts on the faculty training workshops that you attended on [insert Date]?

*Prompts if needed:*

- *What is your perception of relevance and benefit?*

*What did you like, what did you not like, any suggestions, scope of future training?*

1. In your opinion, to what extent this tailored suite of PRiME faculty training workshops are satisfying or attractive to the attendees?
2. Would you be able to implement the workshop learnings?
3. In your perspective and experience, to what extent this tailored suite of PRiME faculty training workshops is likely to be used by medical (dental) educators in Ghana?
4. If these workshops are to be offered regularly, how much demand is likely to exist?

*Prompts if needed:*

- *Who would be the main population of interest?*

1. In your opinion, to what extent this tailored suite of PRiME faculty training workshops can be successfully delivered to medical (dental) educators in both public and private medical schools in Ghana?

*Prompts if needed:*

- *What are the major facilitators for successful delivery?*
- *What are the major obstacles or barriers to successful delivery?*

1. In your opinion, to what extent this tailored suite of PRiME faculty training workshops can be carried out with medical (dental) educators in Ghana using existing means, resources, and circumstances and without outside intervention?

*Prompts if needed:*

- - *What are your thoughts on the availability of human resources?*
  - *What are your thoughts on the availability of material resources?*
  - *What are your thoughts on associate costs?*
  - *What sort of support is required within the Ghanaian context, if any?*

1. In your opinion, to what extent this tailored suite of PRiME faculty training workshops can be integrated into the existing system in Ghana?
2. Is there anything else you would like to highlight that we have not discussed already?
