# Supplementary material for: Acceptability and feasibility of a faculty development programme for medical and dental academics in Ghana
Source: BMC Med Educ. 2026 Feb 21;26:506. doi: 10.1186/s12909-026-08826-3 (PMC13032255; doi:10.1186/s12909-026-08826-3)
Supplement: Supplementary file 3 — Supplementary Material 3. [file 12909_2026_8826_MOESM3_ESM.docx]

**Appendix 3**

Participant responses to Likert-scale questions in the survey.

| Question | Options | Responses per option (%) |
| --- | --- | --- |
| Did you like or dislike this suite of faculty training workshops? | Strongly dislike  Dislike  Neutral (neither like nor dislike)  Like  Strongly like  No response | 0  0  1 (4%)  10 (37%)  16 (60%)  0 |
| How easy was it to engage with these faculty training workshops? | Very difficult  Somewhat difficult  Neutral (neither easy or difficult)  Somewhat easy  Very easy  No response | 0  0  1 (4%)  6 (22%)  16 (59%)  0 |
| How well did the workshops align with your personal values relating to teaching? | Not aligned  A little aligned  Somewhat aligned  Mostly aligned  Completely aligned  No response | 0  0  2 (7%)  17 (63%)  7 (26%)  1 (4%) |
| This suite of faculty training workshops has improved my teaching competencies. | Strongly disagree  Disagree  Neutral (neither agree or disagree)  Agree  Strongly agree  No response | 0  0  4 (15%)  14 (52%)  9 (33%)  0 |
| It is clear to me how these faculty training workshops will help improve my teaching. | Strongly disagree  Disagree  Neutral (neither agree or disagree)  Agree  Strongly agree  No response | 0  0  4 (15%)  11 (41%)  11 (41%)  1 (4%) |
| How confident did you feel about engaging with these faculty training workshops? | Very unconfident  Unconfident  Neutral (neither unconfident or confident)  Confident  Very confident  No response | 0  0  0  3 (11%)  13 (48%)  11 (41%)  0 |
| Engaging in these faculty training workshops interfered with my other priorities. | Strongly disagree  Disagree  Neutral (neither agree or disagree)  Agree  Strongly agree  No response | 2 (7.4%)  9 (33%)  4 (15%)  9 (33%)  3 (11%)  0 |
| Overall, how acceptable was this suite of faculty training workshops to you? | Completely unacceptable  Unacceptable  Neutral (neither unacceptable or acceptable)  Acceptable  Completely acceptable  No response | 0  0  0  2 (7%)  12 (44%)  13 (48%)  0 |
